# Mirtazapine for gastrointestinal and neuropsychological symptoms in older adults with irritable bowel syndrome

**DOI:** 10.1177/17562848241278125

**Published:** 2024-10-24

**Authors:** Ayesha Khan, Raakhi Menon, Brooke Corning, Steven Cohn, Cecil Kumfa, Mukaila Raji

**Affiliations:** Department of Internal Medicine, University of Texas at Medical Branch, 301 University Blvd, 5.138 RS, Galveston, TX 77555-5302, USA; Department of Internal Medicine, University of Texas at Medical Branch, Galveston, TX, USA; Department of Gastroenterology and Hepatology, University of Texas at Medical Branch, Galveston, TX, USA; Department of Gastroenterology and Hepatology, University of Texas at Medical Branch, Galveston, TX, USA; Division of Geriatrics and Palliative Medicine, University of Texas at Medical Branch, Galveston, TX, USA; Division of Geriatrics and Palliative Medicine, University of Texas at Medical Branch, Galveston, TX, USA

**Keywords:** elderly, IBS, mirtazapine, polypharmacy

## Abstract

Irritable bowel syndrome (IBS) is a common and potentially modifiable contributor to excess disability, morbidity, and poor quality of life. Clinical trials of medications for IBS have largely been in younger adults. Yet, a growing number of adults aged 65 and older are living with IBS. No data exist to guide clinicians in the safe and effective use of medications (e.g., anticholinergics, anti-spasmodics, and tricyclic antidepressants (TCA)) for IBS in the geriatric population. These medications—especially anticholinergics and TCAs—carry a high risk of adverse effects (ADE) in older adults because of age-associated decline in drug metabolism and the high prevalence of multiple chronic conditions. Five or more medications (polypharmacy) are frequently used to treat common psychiatric and medical comorbidities of IBS: anxiety, depression, insomnia, migraine headache, diarrhea, nausea, poor appetite, pruritus/skin atopy, and fibromyalgia. These neurological and psychiatric comorbidities reflect shared pathogenic mechanisms and bidirectional crosstalk of high inflammation, alteration of gut microbiota, and dysregulation of multiple gastrointestinal and central nervous system-active neurotransmitters (e.g., serotonin, neuropeptides). Currently, these IBS-associated conditions are treated with multiple medications—which increase the risk of adverse drug–drug interactions. One way to reduce the number of medications used for IBS-associated conditions is the use of one medication that treats many or all of these conditions—Mirtazapine. In this perspective article, we present evidence from basic science, case series, observational and epidemiological studies, clinical studies, and clinical trials supporting mirtazapine, a noradrenergic and specific serotonergic receptor antagonist—with 5-hydroxytryptamine-2 and 3 antagonism, as a potential pharmacotherapeutic intervention for the myriad symptoms and conditions associated with IBS. Specifically, we found evidence of mirtazapine’s role in treating diarrhea, insomnia, migraine headache, nausea, and poor appetite. We propose a large randomized controlled trial to study mirtazapine as a potential one-stop treatment for multiple IBS symptoms, with the potential to reduce polypharmacy and ADEs, especially in the geriatric population.

## Plain language summary

Mirtazapine for gastrointestinal and neuropsychological symptoms in older adults with irritable bowel syndrome

A growing number of adults aged 65 and older are living with irritable bowel syndrome (IBS). Clinical trials of medications for IBS have largely focused on younger adults, leaving a gap in data to guide clinicians in the safe and effective use of these medications for the geriatric population. Many of these medications—especially anticholinergics and tricyclic antidepressants (TCAs)—carry a high risk of adverse effects (ADEs) in older adults. Additionally, polypharmacy, defined as the use of five or more medications, is frequently employed to treat common IBS-associated conditions, including anxiety, depression, insomnia, migraine headaches, diarrhea, nausea, poor appetite, pruritus/skin atopy, and fibromyalgia. One strategy to reduce the number of medications used for IBS-associated conditions is to manage these conditions with a single agent: mirtazapine, a noradrenergic and specific serotonergic receptor antagonist. In this perspective article, we present evidence from basic and clinical studies supporting mirtazapine as a potential therapy for the myriad gastrointestinal and neuropsychological symptoms associated with IBS. Specifically, we found strong evidence for mirtazapine’s role in treating diarrhea, insomnia, anxiety, migraine headaches, and nausea, as well as modest evidence for its effectiveness in treating pruritus/skin atopy and fibromyalgia. We propose a large-scale study on mirtazapine as a potential one-stop treatment for multiple IBS symptoms, with the potential to reduce polypharmacy and ADEs, especially in the geriatric population.

## Introduction

Irritable bowel syndrome (IBS) is a common and potentially modifiable contributor to excess disability, morbidity, and poor quality of life.^1,2^ Predominantly bowel habit-driven, IBS is a pleomorphic disorder with concurrent psychiatric and medical comorbidities including anxiety, depression, insomnia, migraine headache, diarrhea, nausea, poor appetite, pruritus/skin atopy, and fibromyalgia. Clinical trials of pharmacological interventions for IBS have largely focused on younger adults. However, a growing number of adults aged 65 and older are living with IBS,^1,3–12^ reflecting both the aging of patients with IBS and an increase in newly diagnosed older patients. Currently, there are no data to guide clinicians in the safe and effective use of medications (e.g., anticholinergic antispasmodics such as dicyclomine (Bentyl) and tricyclic antidepressants (TCAs) such as amitriptyline) for IBS with diarrhea (IBS-D) predominance in the geriatric population. These medications—especially anticholinergics and TCAs—carry a high risk of adverse effects (ADE) (e.g., confusion, drowsiness, urinary retention) in older adults and are included in the Beers List of potentially inappropriate drugs for the elderly.^13^ Recent AGA guidelines on the use of eluxadoline, rifaximin, alosetron, loperamide, TCAs, and antispasmodics provide little guidance on treating the elderly population with IBS-D.^1^

Similar to the 2022 AGA guidelines, the United European Gastroenterology and European Society for Neurogastroenterology and Motility also emphasized the complexity of choice and the side effect profiles of various pharmacologic agents for IBS, including antispasmodics, the 5-hydroxytryptamine (5-HT)3 antagonist alosetron, and eluxadoline with mixed opioid activity.^14^ Newer drugs have been tested primarily in younger populations. Other medications used in IBS address the common psychiatric conditions and medical comorbidities of IBS: anxiety, depression, insomnia, migraine headache, diarrhea, nausea, poor appetite, pruritus/skin atopy, and fibromyalgia. These neurological and psychiatric comorbidities reflect shared pathogenic mechanisms and bidirectional crosstalk of high inflammation, alteration of gut microbiota, and dysregulation of multiple gastrointestinal (GI) and central nervous system-active neurotransmitters (e.g., serotonin, neuropeptides).^3–12^

Currently, each of these IBS-associated conditions is treated with different medications, leading to polypharmacy, a high risk of adverse drug–drug interactions, and increased healthcare costs.^2^ The risks of adverse drug effects are especially high in older adults, who have an age-associated decline in drug metabolism and clearance, and a high prevalence of multiple chronic conditions that require multiple medications.^15^ One way to address the issue of multiple medications for multiple IBS-associated conditions is to use a single medication that treats many or all of these conditions.

In this perspective article, we present evidence from basic science, case series, observational and epidemiological studies, clinical studies, and clinical trials supporting mirtazapine, a noradrenergic and specific serotonergic receptor antagonist, as a one-stop pharmacotherapeutic intervention for the myriad symptoms and conditions associated with IBS. Limited data exist on the incidence of IBS, particularly IBS-D, within the elderly population. The last epidemiological study, conducted in 2005, reported that 10%–20% of elderly adults exhibit symptoms consistent with IBS, without specifying the subtype. This lack of specificity is partly due to IBS being underdiagnosed in this demographic, influenced by other health comorbidities, polypharmacy, and the incidence of GI cancers.^16^ A special focus of our review is on IBS-D—a highly prevalent type in the geriatric population for which mirtazapine has shown significant evidence for the relief of diarrhea and other comorbidities—anxiety, depression, insomnia, migraine headache, diarrhea, nausea, poor appetite, pruritus/skin atopy, and fibromyalgia.

### Methods

A scoping literature review of research studies published from 1990 to 2023 was conducted through PubMed using the following medical subject heading search terms: mirtazapine, and subheadings including headache/migraine, anxiety and depression, insomnia, diarrhea, nausea/vomiting, poor appetite and weight loss, pruritus/skin atopy and fibromyalgia, or chronic pain. Studies included basic science research, case series, observational and epidemiological studies, clinical studies, and clinical trials. A total of 58 English-language peer-reviewed articles were included. Exclusion criteria were non-English articles and non-peer-reviewed articles.

## Background

IBS is defined by the Rome IV Criteria as recurrent abdominal pain associated with two or more of the following symptoms: related to defecation, change in the frequency of stool, and change in the consistency of stool, occurring at least 1 day per week in the last 3 months.^15^ IBS is categorized into four subtypes: IBS with constipation, IBS-D, mixed, and unclassified.^17^ The estimated prevalence of IBS, according to the Rome IV Criteria, ranges from 4.7% to 5.3%.^18^ Most epidemiological studies published on IBS are derived from patient-reported symptom surveys and often exclude the geriatric population. In a survey conducted in Olmsted County, MN, focusing on elderly community residents aged 65–93, a higher prevalence of diarrhea (9.6%–14.2%) was observed.^19^

Growing evidence supports IBS as a multisystemic condition with substantial neuropsychiatric and dermatological components, reflecting bidirectionality in the gut–brain, gut–lung, and gut–skin axes—a reflection of roles of immune dysfunction, mast cell activation, and elevated serotonin levels.^20–24^ Serotonin plays an important role in the enteric nervous system (ENS) and is a key neuromodulator in the gut–brain axis.^19–25^ The main receptors involved are 5-HT3, 5-HT4, and 5-HT1b. Serotonin release from enterochromaffin cells stimulates 5-HT3 receptors, resulting in nausea, bloating, and a sensation of fullness. 5-HT4 receptors work with 5-HT1b receptors to increase GI motility and intestinal secretions.^19–25^ Increased serotonin plasma levels have been reported in IBS-D, whereas the opposite has been reported in constipation-predominant IBS.^26–32^ Consequently, anxiolytics and antidepressants are commonly employed in symptom management.

There is substantial evidence supporting the efficacy of serotonin type 3 (5-HT3) receptor antagonists in treating patients with IBS-D. Mirtazapine, an atypical antidepressant drug and a potent serotonin type 3 (5-HT3) and type 2 (5-HT2) receptor antagonist, has demonstrated benefits in reducing symptom severity in this specific subset of patients.

## Mechanism of actions of mirtazapine underlying its roles in IBS

The expression of certain receptors can be upregulated in IBS, specifically histamine H1 and H2 receptors. Serotonin is an important neuromodulator in the gut–brain axis, playing major roles in modulating the functions of the ENS and GI smooth muscles and vasculature via the 5-HT1A, 5-HT1C, 5-HT2, 5-HT3, and 5-HT4 receptors, which are abundantly located throughout the GI system.^27–30^ Patients with IBS-D may have substantial dysregulation of various serotonin receptors and sub-receptors and impairment in serotonin uptake and release.^27–31^ For example, stimulating 5-HT2 receptors in the gut leads to contraction of GI smooth muscle, whereas the opposite effect occurs when 5-HT1 receptors are stimulated.^27^ Mirtazapine enhances the function of 5-HT1A and 5-HT1C receptors while blocking 5-HT2 and 5-HT3 receptors.^27–35^ In particular, 5-HT3 receptors, which play a major role in gut function and IBS pathogenesis, are found in enteric motor neurons, peripheral afferents, and the vomiting center.^20–28^ Blocking 5-HT3 receptors reduces pain and decreases colonic transit and small intestinal secretion, thereby decreasing diarrhea.^27–35^

**Figure 1.**
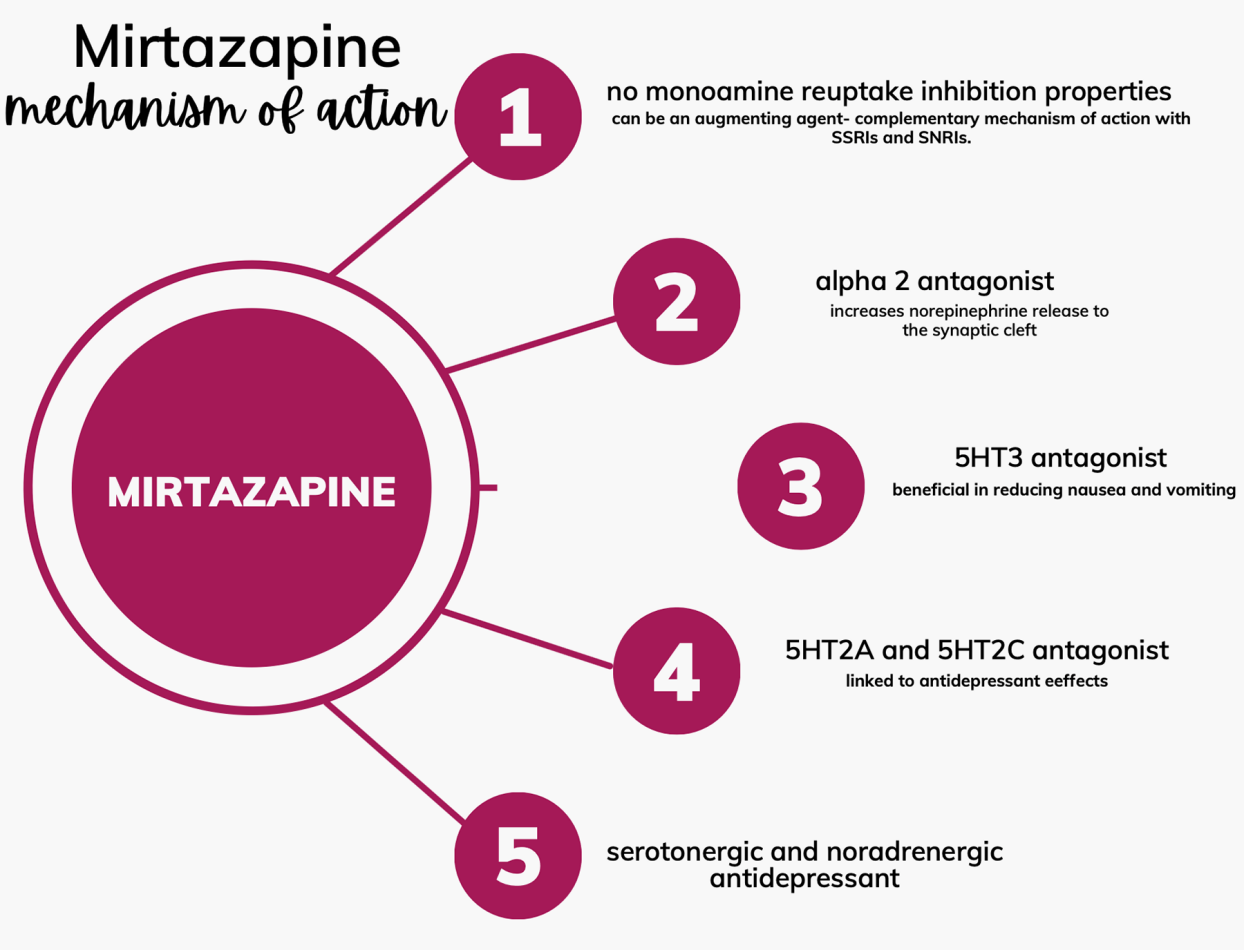
Mirtazapine mechanism of action.

Mirtazapine is an antidepressant and antianxiety agent that acts by blocking presynaptic α2-adrenergic autoreceptors and α2-adrenergic heteroreceptors and by antagonizing 5-HT2 and 5-HT3 receptors, enhancing the effects of 5-HT1A-mediated serotonergic transmission.^31,34^ Mirtazapine also has effects on other subtypes of 5-HT1 receptors. It has a high affinity for central and peripheral H1 receptors and acts as a potent antagonist of H1 receptors (Figure 1).^31^ Commonly reported side effects include dry mouth, somnolence, hyperphagia, increased appetite, body weight gain, and transient somnolence, which are associated with the antihistaminic (H1) activity of mirtazapine at low doses.^35^ Mirtazapine is FDA approved for major depressive disorder, with the advantages of a faster onset of action than selective serotonin reuptake inhibitors (SSRIs).^36,37^ It is commonly used off-label to treat comorbid anxiety, insomnia, and appetite loss, especially when they coexist with depression.

Thus, we discuss below the potential for off-label use of mirtazapine for the myriad GI and neuropsychological symptoms in older adults with IBS, using evidence from studies on the use of mirtazapine in other related conditions with symptoms similar to IBS.

Mirtazapine undergoes extensive liver metabolism primarily via the cytochrome P450 isoenzymes CYP1A2, CYP2D6, and CYP3A4. Achieving steady-state concentrations takes about 4 days in adults and 6 days in the elderly when dosed once daily. In vitro studies indicate that mirtazapine is unlikely to result in clinically significant drug–drug interactions. Common ADEs include dry mouth, sedation, increased appetite, and weight gain. Notably, unlike SSRIs, mirtazapine does not cause sexual dysfunction.^38,39^

Mirtazapine is less likely to cause hypertension, tachycardia, and tremor compared to TCAs. Compared to SSRIs, mirtazapine is more likely to cause weight gain, increased appetite, salivation, somnolence, and fatigue, but less likely to cause flatulence, sweating, sexual dysfunction, tremor, nausea, vomiting, sleep disturbances, and diarrhea. Compared to the serotonin–noradrenaline reuptake inhibitor venlafaxine, mirtazapine is more likely to cause fatigue but less likely to cause sleep disturbances, sweating, and constipation. Compared to trazodone, mirtazapine is more likely to cause weight gain and increased appetite. Approximately 70% of patients on mirtazapine experienced at least one adverse event, similar to other antidepressants.^38,39^

## Clinical applications for symptom control—data from clinical studies

### General IBS symptoms and quality of life

Preliminary data points to the potential role of mirtazapine in relieving the abdominal cramps and pain associated with IBS.^35^ Khalilian and colleagues conducted a randomized double-blind trial among 67 patients meeting Rome IV criteria for IBS-D, with randomization to a mirtazapine treatment group (*n* = 34) or a placebo treatment group (*n* = 33). They found that mirtazapine had higher efficacy in reducing the severity of IBS symptoms (*p*-value = 0.002) and the severity of all dairy-derived symptoms compared to the placebo. In addition, patients in the mirtazapine group had significantly improved quality of life and anxiety symptoms compared to patients in the placebo group (*p*-value = 0.005).^31^

### Migraine/headache

Worldwide, numerous investigations have revealed a substantial connection between migraines and IBS. Analyzing data from a prominent US health plan, a cohort study explored the prevalence of migraines among individuals with IBS, revealing that those with IBS faced a 60% elevated risk of experiencing migraines compared to those without IBS.^40^

Dysfunction in central serotonin (5-HT) neurotransmission is central to migraine pathology. Chronic low levels of 5-HT contribute to migraine development, while sudden surges in 5-HT release trigger migraine attacks. In addition, migraine sufferers exhibit hypersensitivity to nitric oxide-releasing agents like 5-HT and histamine, which can lead to cerebral vasodilation, neurogenic inflammation, and trigeminovascular activation.^41^ The mechanisms behind mirtazapine’s efficacy in treating migraines can be understood in two ways. The reduced occurrence of migraines with mirtazapine is attributed to its blockade of 5-HT2 (particularly 5-HT2B) and histamine receptors, which are implicated in migraine initiation.^41^ In addition, mirtazapine’s activation of 5-HT1 receptors, including 5-HT1B, 5-HT1D, and 5-HT1F, may alleviate migraines by constricting dilated cerebral vessels and reducing neurogenic inflammation.^41^ However, at higher doses, the activation of histamine may contribute to migraine recurrence.

A study conducted by Bendtsen et al. involved 24 individuals without depression, suffering from chronic tension-type headaches, who participated in a randomized, double-blind, placebo-controlled, crossover trial. Each participant received either mirtazapine at doses of 15–30 mg/day or a placebo for 8 weeks, with a 2-week wash-out period in between. The study found that during the treatment with mirtazapine, the primary efficacy measure, area-under-the-headache curve (AUC; calculated by multiplying duration with intensity), was significantly lower compared to the placebo.^33^ Furthermore, mirtazapine demonstrated a reduction in secondary efficacy variables such as headache frequency, duration, and intensity.^42^

In a randomized, double-blind, placebo-controlled crossover trial with 24 patients suffering from chronic tension-type headaches, mirtazapine was found to significantly reduce headache severity, performing comparably to amitriptyline. A subsequent double-blind, placebo-controlled parallel trial involving 93 patients compared mirtazapine, ibuprofen, and their combination. It revealed that low-dose mirtazapine alone could reduce headache severity by 20%, demonstrating a clear dose–response relationship in terms of both efficacy and tolerance.^43,44^

### Anxiety and depression

Mirtazapine has shown promise in managing symptoms of depression and anxiety associated with IBS. Serotonin reuptake transporter transcription is decreased in patients with IBS, leading to increased serotonin through increased neuronal uptake or decreased production, which, in turn, induces anxiety.^33,45^ Mirtazapine acts as an α2-adrenergic autoreceptor and α2-adrenergic heteroreceptor blocker and antagonizes 5HT2 and 5HT3 receptors, enhancing the effects of 5HT1A-mediated serotonergic transmission.^31,34^ In a case report by Spiegel et al., a patient with comorbid panic disorder, depression, and mixed-type IBS was successfully treated with mirtazapine, showing improvement in diarrhea, constipation, and psychopathological symptoms.^33^ Mirtazapine (15–45 mg) is similar in efficacy and safety compared to amitriptyline (30–90 mg) in treating depressed patients aged 60–85 years.^46–51^ Mirtazapine has been used in many clinical trials to significantly help with depression and is widely accepted as an effective FDA-approved antidepressant.^46^

A 12-week open-label study by Gambi et al.^45^ showed mirtazapine as a promising drug for generalized anxiety disorder. The outcomes measured were changes from baseline in the total score on the Hamilton Rating Scale for Anxiety (HAM-A) and the Clinical Global Impression of Improvement (CGI-I) rated at the endpoint. Patients with a reduction of 50% or more on the HAM-A total score and a CGI-I score of 1 or 2 at the endpoint were considered responders to treatment; a 79.5% response rate was achieved.^48^

Geriatric depression has been specifically studied with the use of mirtazapine. Multiple studies found that mirtazapine improves depression, insomnia, anxiety, and somatic symptoms.^38,39^ In a comparison study of depressed subjects aged 65 years or older, both mirtazapine and paroxetine were effective; however, mirtazapine demonstrated a faster response time of 26 days compared to 40 days for paroxetine and was associated with a greater reduction in anxiety/somatization and sleep disturbance scores.^51^

Patients with IBS have a higher prevalence of comorbid anxiety and depression. In a systematic review, patients with IBS had significantly higher anxiety and depression levels than controls (standardized mean difference (SMD) = 0.76, 95% confidence interval (CI): 0.47–0.69, *p* < 0.01, *I*^2^ = 81.7% and SMD = 0.80, 95% CI: 0.42–1.19, *p* < 0.01, *I*^2^ = 90.7%).^49^

### Insomnia

The prevalence of sleep disorders comorbid with IBS is 37.6% (95% CI: 31.4%–44.3%) based on a meta-analysis.^52^ Mirtazapine’s role as a potent antagonist of central and peripheral H1 receptors has been beneficial in treating insomnia.^31,35^ In addition, speculation exists regarding mirtazapine’s action at the 5-HT2 receptor in the treatment of insomnia.^53^ In a 2-week trial involving doses of 15 and 30 mg of mirtazapine in 130 depressed patients with insomnia, improvements in symptoms were noted at both doses.^54^ Initially, a 10% incidence of somnolence decreased as the trial progressed, suggesting rapid development of tolerance to the sleep-inducing effect of the H1 antagonist.^54^ In a double-blind study of 19 depressed patients with insomnia comparing mirtazapine with fluoxetine, the mirtazapine group showed improvements in total sleep time and sleep latency; however, statistical significance compared to fluoxetine was not achieved.^53^

### Diarrhea

The role of mirtazapine in treating diarrhea has been extensively studied. IBS-associated diarrhea in elderly patients does not differ significantly from younger patients. A study using a rat model included 20 colonic-sensitized rats and 20 matched controls.^45^ Visceral sensitivity during colorectal distension was assessed using abdominal electromyogram measurements.^45^ Mirtazapine dose dependently affected visceral hypersensitivity in colonic-sensitized rats and marginally improved delayed gastric emptying while slightly delaying small intestinal transit.^45^

5-HT dysfunction in the gut can affect intestinal motor and secretory function, potentially leading to either constipation or diarrhea in IBS.^31^ An Iranian double-blind, randomized, placebo-controlled study investigated the effects of mirtazapine in IBS-D patients.^31^ Outcomes were based on changes in the total IBS symptom severity score (IBS-SSS), Hospital Anxiety and Depression Scale score (HADS), and IBS Quality of Life (IBS-QoL).^31^ The mean age of patients in the mirtazapine and placebo groups was 44.41 (±11.25) and 43.45 (±10.35) years, respectively. Patients in the mirtazapine group showed significantly greater improvement in IBS-SSS score compared to those in the placebo group (−89.76 ± 71.60 vs −34.73 ± 66.91; *p*-value = 0.002).^31^ Mirtazapine also reduced the average number of bowel movements from 2.30 ± 0.69 (prior to starting treatment) to 1.83 ± 0.43 (at the end of the trial).^31^ Patients noted improvements in depression symptoms compared to placebo-treated subjects.^31^ Furthermore, there was a significant improvement in IBS-QoL score in the mirtazapine group compared to the placebo group; the IBS-QoL score increased from 45.32 ± 17.18 at baseline to 70.52 ± 16.55 at week 8 post-treatment in the mirtazapine group, compared to an increase from 53.11 ± 16.34 to 62.68 ± 14.25 in the placebo group; *p*-value = 0.04.^31^ Mirtazapine improved stool consistency, decreased stool frequency and urgency, reduced abdominal pain scores, and increased the number of days without bowel urgency, pain, and diarrhea.^31^

An open-label study at a tertiary referral center included 19 patients with a mean age of 39 years with IBS-D. Patients started on 15 mg daily and increased to 30 mg of mirtazapine. Outcomes were measured using the IBS-SSS, HADS, and diary-derived symptom scores (diarrhea, pain, urgency) post-treatment compared to baseline. Significant reductions in symptom scores for abdominal pain, urgency, diarrhea, and bloating were observed (all *p* ⩽ 0.01).^34^

### Nausea/vomiting

Mirtazapine exerts antagonistic effects on the 5-HT3 receptor, contributing to its antiemetic properties.^55,56^ It has been established as an effective treatment for nausea and vomiting. In a randomized placebo-controlled trial, mirtazapine demonstrated efficacy when administered 1 h prior to orthopedic surgery.^29,30^ The incidence of postoperative nausea and vomiting was significantly lower in the mirtazapine group compared to the placebo group.^46,57^ In a study by Malamood et al.^55^ mirtazapine also significantly reduced nausea and vomiting in patients with refractory gastroparesis at 2 and 4 weeks post-treatment.^58^

### Poor appetite and weight loss

Antidepressants are known to influence weight gain and appetite stimulation, which in certain patient populations may be considered a side effect but in elderly individuals can be a beneficial effect of treatment.^59^ Atypical antidepressants like mirtazapine have demonstrated the benefit of not only improving mood but also promoting weight gain. Studies indicate an average weight gain of 1.74–2.59 kg with long-term (>4 months) and short-term (4–12 weeks) use of mirtazapine, respectively.^55,60^ The precise mechanism by which mirtazapine restores appetite is not fully understood, although it may involve acceleration of gastric emptying possibly due to 5-HT2C antagonism/inverse agonism.^55,61^ In addition, the antihistaminic effects of mirtazapine could contribute to increased appetite.^55^

In a study involving women diagnosed with depressive episodes, monotherapy with mirtazapine over a 6-week period significantly increased body weight, body fat mass, and leptin concentration.^20^ An open-label clinical trial in healthy men showed that mirtazapine increased hunger and appetite for sweets, accompanied by a preference for carbohydrate substrate and increased insulin and C-peptide release in response to meals.^62^

Furthermore, an open-label single-institution phase II trial of mirtazapine in non-depressed patients with cachexia and anorexia related to underlying cancer reported that 24% of patients gained at least 1 kg after 4 weeks of therapy. Patients also experienced reductions in fatigue, nausea, and early satiety, which correlated with the observed weight gain.^55,63^ These findings underscore mirtazapine’s potential role in addressing weight loss and poor appetite associated with IBS.

### Pruritus/skin atopy

IBS has been noted to be associated with atopic dermatitis in patients.^20^ The literature extensively describes the bidirectional association between IBS and pruritus/skin atopy.^65–67^

Mirtazapine has demonstrated benefits in the treatment of pruritus. An 8-week prospective, open-label, cross-over randomized clinical trial was conducted on 77 hemodialysis patients with chronic pruritus. Patients were treated with mirtazapine (15 mg per day) or gabapentin (100 mg per day) for 2 weeks in each treatment period. Sixty-one patients completed both treatment periods, and improvement in pruritus severity was significantly greater during the mirtazapine treatment period compared to the gabapentin treatment period (*p* < 0.001).^63^ Mirtazapine’s mechanism involves antagonizing α2-adrenergic receptors, making it effective in managing chronic pruritus refractory to typical first-line therapies.^64,65^

### Fibromyalgia

Mirtazapine has been explored as a treatment option for fibromyalgia due to its blockade of 5-HT2 and 5-HT3 receptors.^66^ In a 6-week open-label trial involving 29 patients with fibromyalgia who received mirtazapine at doses of 15–30 mg/day, 54% of the 26 patients who completed the study reported a clinically significant reduction in pain intensity and their mean weekly acetaminophen dosage.^46,66^

Mirtazapine’s antinociceptive effects are mediated through serotonergic, noradrenergic, and opioid mechanisms.^35,67^ A post-marketing surveillance study conducted over 6 weeks in 239 psychiatric and neurological outpatient facilities demonstrated that patients with chronic pain and concurrent depression experienced statistically significant reductions in pain from baseline to endpoint with the use of mirtazapine.^35^

## Clinical implications and conclusion

IBS-D presents a significant therapeutic challenge across all age groups. In older adults particularly, the wide array of GI and neuropsychiatric symptoms associated with IBS can profoundly impact social and functional quality of life. This literature review examines several studies on the use of mirtazapine for conditions with symptoms akin to IBS, highlighting its potential benefits.

While other medications such as anticholinergics and TCAs can address multiple GI and neuropsychological symptoms of IBS-D, mirtazapine offers a comprehensive strategy that may reduce polypharmacy and associated ADEs. We present both basic science and clinical data supporting mirtazapine’s role in alleviating various co-occurring symptoms—such as depression and insomnia—that are common in IBS-D.

Currently, neither US nor European IBS guidelines advocate for a single-medication approach to manage multiple comorbid symptoms of IBS, underscoring the importance of raising awareness about mirtazapine’s potential in this context. Such an approach could mitigate polypharmacy, minimize adverse drug interactions, and potentially reduce costs. We advocate for large randomized controlled trials, especially in older patients with IBS, to rigorously assess the efficacy and effectiveness of mirtazapine in treating IBS.

## Declarations

**Ethics approval and consent to participate:** Not applicable.

**Consent for publication:** Not applicable.

**Availability of data and materials:** Not applicable.
